# Corn Cobs

**Published:** 1859-10

**Authors:** 


### CORN COBS.

Some fourteen years ago, while passing along the street, we stepped into a gentleman's furnishing store to inquire for a large umbrella. The stock was small and there were none of even medium size. We excused ourselves from a purchase, saying they were not quite as large as we desired.

"They are not large enough for a whole family," responded the storekeeper. Such a reply did not dispose us to trade; so we passed on and obtained one to our liking, which we have to this day, doing good service, with occasional recoverings.

This man was a "*Corn Cob*," too rough to deal with; and, during a residence of seven or eight years afterwards, passing his fine assortment daily, we never entered his store again, not choosing to put ourselves in the way of an incivility.

A popular old Quaker, in the long course of years, maintained a large custom in Philadelphia, and grew very rich without parade or show, or any indication of being in a hurry; others were constantly changing their customers, but the same class of persons, through changing times, stuck to him. It seemed that if a citizen ever made a purchase of him once, he always found his way back again. On being inquired of one day as to what he attributed his extraordinary success, he replied "*Civility*, CIVILITY."

His articles were always good, always full measure, and a disposition to oblige, pervaded his whole character.

We have all heard of a New York incident. An aged lady of wealth called at a store in Broadway to search for a particular article of goods, but, after turning over a large quantity, she was not suited, although the clerk was evidently anxious to make a sale. But his pains were unavailing, she left the store with the purchase of some trifling article of a few cents value. Nothing incommoded, he put it up promptly and accompanied her to the door, pleasantly remarking, that he hoped when she called again he would be better supplied with goods to her liking, and he thought no more of it.

Some months later this lady died, and surprised him with a handsome legacy. His unaffected courtesy, it seems, made a deep impression on her mind, and she adopted this method of rewarding it.

A want of civility is painfully felt in most places of business in New York; it is especially observable in the clerks of our northern steamboats, railway offices and banks, public and private. In striking contrast is all this with what we have observed in Paris. At the stores, money exchanges, and railway stations you are waited on by women, and in handing you your ticket or change there is a cordial "thank you," as if you had really made a present, or conferred a special favor. We, in our American boorishness, may talk as much as we please about the hollowness of French politeness; even if it is so, if leaves upon the mind of a stranger or traveller a very agreeable impression, a kind of make-up for the many unavoidable roughnesses of travel. A woman's smile is always agreeable, even if you have some spiteful conjecture that it may be as empty as the air cushion you sit upon. "*Nothing in it*" there may be, but it is comfortable for all that.

None but a natural "*Corn Cob*" sees only hollowness in every act of unpurchased civility. Get out of the way you ugly porcupine! and dwell you among bears and the men and women made like them, by the disappointments of life—such disappointments as always will fall to the lot of those who cherish anticipations which they never merited, and whose spiteful interpretations of the little civilities of the better-hearted of our kind, only mirror the motives which prompt an occasional counterfeit on their own part. Civility is a virtue which grows by its practice, and is constantly reproducing itself, while its rewards are always sweet, always elevating, and sometimes large; it has an important agency in soothing the rugged pathway of life; it plants a flower where else would grow a thorn; it lightens with smiles the face on which sadness has made a resting place, and in doing this adds to human happiness and human health.

